# Elevated Interleukin-6 Is Associated with an Increased Risk of Long-Term Arteriovenous Fistula Failure for Dialysis

**DOI:** 10.3390/jcm14020488

**Published:** 2025-01-14

**Authors:** Claudiu Constantin Ciucanu, Alexandru Mureșan, Elena Florea, Bartus Réka, Adrian Vasile Mureșan, Ludovic-Alexandru Szanto, Eliza-Mihaela Arbănași, Ioan Hosu, Eliza Russu, Emil-Marian Arbănași

**Affiliations:** 1 Department of Vascular Surgery, George Emil Palade University of Medicine, Pharmacy, Science and Technology of Targu Mures, 540139 Targu Mures, Romania; claudiu.ciucanu@umfst.ro (C.C.C.); adrian.muresan@umfst.ro (A.V.M.); eliza.russu@umfst.ro (E.R.); emil.arbanasi@umfst.ro (E.-M.A.); 2 Clinic of Vascular Surgery, Mures County Emergency Hospital, 540136 Targu Mures, Romania; alex.muresan99@yahoo.ro (A.M.); dr.florea.elena@gmail.com (E.F.); szanto.ludovic@gmail.com (L.-A.S.); 3 Doctoral School of Medicine and Pharmacy, George Emil Palade University of Medicine, Pharmacy, Science and Technology of Targu Mures, 540139 Targu Mures, Romania; arbanasi.eliza@gmail.com; 4 Regenerative Medicine Laboratory, Centre for Advanced Medical and Pharmaceutical Research (CCAMF), George Emil Palade University of Medicine, Pharmacy, Science and Technology of Targu Mures, 540139 Targu Mures, Romania; 5 Department of Nephrology, Mureș County Emergency Hospital, 540136 Targu Mures, Romania; ioan.hosu@umfst.ro

**Keywords:** arteriovenous fistula, AVF, vascular surgery, interleukin-6, dialysis, end-stage kidney disease, vascular access

## Abstract

**Background/Objectives**: The autologous arteriovenous fistula (AVF) is the preferred choice for vascular access in patients with end-stage kidney disease (ESKD) undergoing maintenance hemodialysis. However, in the long term, the primary patency of AVF is suboptimal, with an AVF failure of approximately 30% in one year. The aim of this study is to examine how the pre-operative baseline levels of interleukin-6 (IL-6) affect long-term AVF failure. **Methods**: This retrospective, observational study involves ESKD patients admitted to the Vascular Surgery Clinic for AVF creation from January 2020 to December 2023. Ultimately, a total of 91 patients whose AVFs matured and began dialysis were enrolled. Prior to surgery, each patient underwent a thorough blood sample collection, with IL-6 levels assessed. The patients were categorized into two groups: those with functioning AVFs and those with dysfunctional AVFs. Their progress was monitored via a review of medical records, telephone interviews, or direct contact. Following the surgery, patients were observed for an average of 1.53 ± 0.94 years. **Results**: During the follow-up, patients who experienced AVF failure had a higher incidence of diabetes mellitus (*p* = 0.019) and active smoking (*p* = 0.012), as well as higher levels of IL-6 (*p* < 0.001). At ROC analysis, we found a strong association between IL-6 value and AVF failure (AUC: 0.814, *p* < 0.001), with an optimal cut-off value of 7.08 (76.5% Sensitivity and 79.7% Specificity). Furthermore, at the survival curve Kaplan-Meier analysis, we observed a higher occurrence of AVF failure in patients with baseline IL-6 values above the median (*p* = 0.004), in tertile 3 (*p* = 0.002), and above the optimal cut-off value (*p* < 0.001). At cox-regression analysis, elevated baseline IL-6 levels are associated with AVF Failure (HR: 2.23, *p* < 0.001). **Conclusions**: In the current study, we demonstrated that elevated IL-6 levels at baseline are associated with long-term AVF failure, independent of age, sex, and cardiovascular risk factors.

## 1. Introduction

Chronic kidney disease (CKD) is the ninth leading cause of death worldwide and is a burden on both the patient and the healthcare system [1]. Patients diagnosed with end-stage kidney disease (ESKD) require kidney replacement therapy (KRT), such as hemodialysis and peritoneal dialysis, until they can benefit from a kidney transplant [2]. Due to the limited number of donors and the advanced age of ESKD patients, most patients have to choose chronic hemodialysis [3].

Among the optimal choices for vascular access for hemodialysis, the arteriovenous fistula (AVF) is the preferred option, followed by the arteriovenous graft fistula (AVG) and central venous catheter (CVC) for dialysis [4,5,6,7]. However, in the long term, the primary and secondary patency of AVF is suboptimal according to the guidelines of the European Society of Vascular Surgery (ESVS) [4]. They report an incidence of secondary patency of AVF ranging from 42% to 83% at one year for radio-cephalic arteriovenous fistula (RC-AVF), and from 66% to 89% for brachio-cephalic arteriovenous fistula (BC-AVF) [4]. For this reason, maintaining the patency of the fistula and avoiding the complications that may occur at the AVF level are crucial.

Although the pathophysiological mechanisms underlying the failure of an arteriovenous fistula have not been fully elucidated, the pivotal role is attributed to inflammation resulting from endothelial injury caused by increased shear stress at the level of the venous wall, which in time will trigger a process of cell proliferation associated with intimal hyperplasia and ultimately will lead to AVF dysfunction [8,9,10,11]. Consequently, interleukin-6 (IL-6) is recognized as a multifunctional cytokine that activates monocytes and macrophages, serving a pivotal role in inflammation and tissue damage, as well as cell activation and proliferation [12]. Furthermore, IL-6 is one of the most potent pro-inflammatory cytokines, initiating the acute phase response in hepatocytes and stimulating the synthesis of C-reactive protein (CRP) [13,14,15].

The aim of this study is to examine how the pre-operative baseline levels of IL-6 affect long-term AVF failure. Additionally, we will investigate the associations between demographic data, comorbidities, risk factors, pre-operative vascular mapping, and AVF failure.

## 2. Materials and Methods

### 2.1. Study Design

The current study is a retrospective and observational study. It includes all patients diagnosed with ESKD who were admitted to the Vascular Surgery Clinic of the Emergency County Hospital of Targu Mures, Romania, between January 2020 and December 2023. Patients admitted to the hospital for reasons unrelated to AVF procedure, those with active tumor status (patients undergoing oncological treatment at the time of enrollment in the study), individuals with hematological diseases, patients for whom IL-6 levels were not assessed preoperatively, and patients lost to follow-up were excluded from the study. Moreover, we excluded patients whose AVF did not fulfill the maturation criteria within 4 to 8 weeks postoperatively and for whom chronic dialysis had not been initiated at the AVF level. After applying these criteria, 91 out of 223 patients were enrolled in the study. These patients were then divided into two groups: those with patent AVF and those with dysfunctional AVF. Additionally, every AVF was surgically created using side-to-end anastomosis, and all procedures were conducted by the same vascular surgeon. The patients’ progress was tracked through medical record review, telephone interviews, or direct patient contact.

### 2.2. Data Collection

Demographic and clinical data of the patients were collected at the time of AVF creation from the hospital’s electronic database. As for comorbidities, we included hypertension, atrial fibrillation (AF), ischemic heart disease (IHD), history of myocardial infarction (MI), diabetes mellitus (DM), cerebrovascular accident (CVA), peripheral arterial disease (PAD), tobacco, and obesity. Blood tests (hemoglobin, hematocrit, neutrophil count, lymphocyte count, monocyte count, platelet count, glucose level, cholesterol, and triglyceride level) and IL-6 levels were measured before surgery. Regarding the type of AVF performed, we enrolled patients with RC-AVF, BC-AVF, or brachio-basilic AVF (BB-AVF), as well as whether the AVF procedure was performed at the level of the dominant or non-dominant upper limb.

### 2.3. Pre-Operative Vascular Mapping

Preoperatively, each patient benefited from vascular ultrasound mapping, during which we determined the site of the AVF creation and the arterial and venous diameter. The criteria for selecting the AVF site were those recommended by the ESVS guideline: for RC-AVF, a minimum diameter of 2 mm of the artery and vein, respectively; for BC-AVF and for BB-AVF, a vascular diameter of at least 3 mm [4].

### 2.4. Follow-Up

The study’s primary endpoint, the AVF failure rate, is defined as the inability to use the AVF for dialysis due to severe blood vessel stenosis or acute thrombosis. We monitored the patients’ progress by reviewing their medical records, conducting telephone interviews, and directly contacting the patients. Additionally, we requested dialysis centers to provide updates on the condition and functioning of the patient’s AVF. We collected information on all patients up to 1 July 2024.

### 2.5. Statistical Analysis

SPSS for Mac OS version 28.0.1.0 was used for statistical analysis (SPSS, Inc., Chicago, IL, USA). Laboratory data and pre-operative vascular mapping information are presented as median (quartile 1-quartile 3). We conducted the Chi-square test to compare the differences between the dichotomous variables. We used the Mann–Whitney and Student’s *t*-test to assess variations in continuous variables. The appropriate IL-6 cut-off value was determined using ROC curve analysis based on the Youden index. We conducted multivariate Cox proportional hazard analyses to find independent predictors of AVF failure. We expressed the hazard ratio (HR) per 1 standard deviation increase in the baseline for IL-6 values. Additionally, we used three different adjustment models to assess the associations between IL-6 and AVF failure. Thus, Model 1 remains unadjusted; Model 2 includes age and sex; and Model 3 includes age, sex, and cardiovascular risk factors (DM, hypertension, IHD, and PAD). We used Kaplan–Meier survival curves to illustrate the unadjusted relationship between IL-6 and long-term AVF failure based on median, tertiles, and cut-off values. The Log Rank test was used to compare the curves. All tests were two-tailed, and a *p*-value less than 0.05 was considered statistically significant.

## 3. Results

In the current study, we included 91 patients with an average age of 62.93 ± 13.28, among whom 53.85% were male. Over the long term, 17 patients experienced AVF failure. The most common comorbidities were hypertension in 91.21% of patients, IHD in 63.74% of patients, and DM in 39.56% of patients (Table 1). Regarding AVF type, we performed 41 RC-AVF (45.05%), 39 BC-AVF (42.86%), and 11 BB-AVF (12.09%), with 16.48% performed on the dominant upper limb. Moreover, the patients were monitored for an average of 1.53 ± 0.94 years after the surgery (Table 1).

During the follow-up, patients who experienced AVF failure were found to have a higher incidence of diabetes mellitus (64.71% vs. 33.78%, *p* = 0.019) and active smoking (35.29% vs. 10.81%, *p* = 0.012). Additionally, they showed higher levels of white blood cells (*p* = 0.008), glucose (*p* = 0.007), neutrophil count (*p* = 0.023), and platelet count (*p* = 0.048), as well as higher levels of IL-6 (*p* < 0.001) (Table 1). However, no differences were observed in pre-operative vascular diameters, the type of AVF, or the use of CVC for dialysis at the time of AVF creation (Table 1).

**Figure 1:**
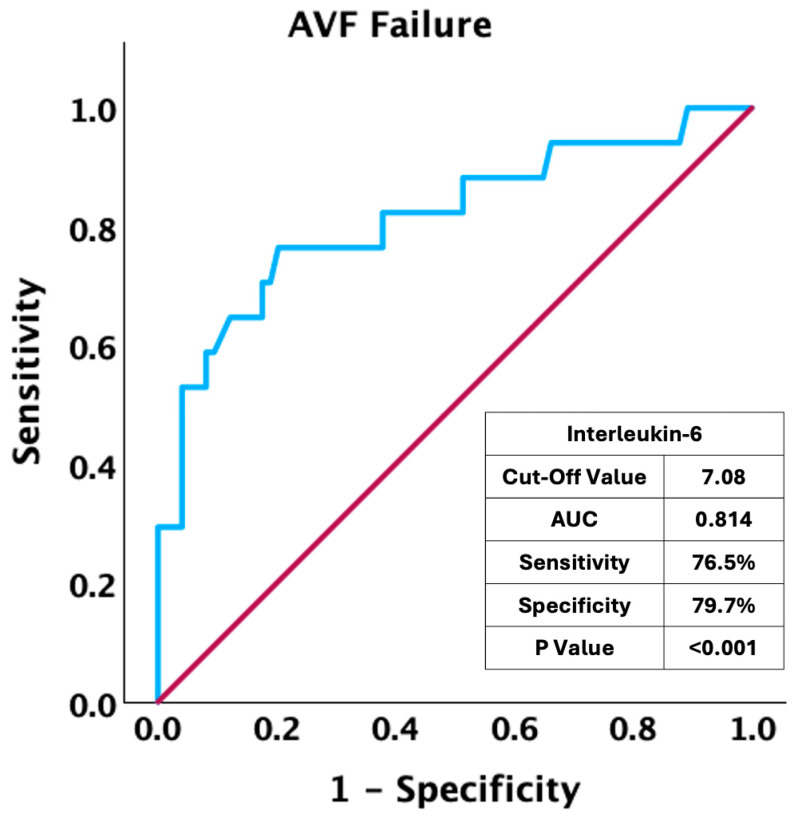
ROC curve analysis: association of interleukin-6 values at baseline and AVF failure.

We performed ROC analysis on IL-6 values to determine the association between baseline levels of this inflammatory biomarker and long-term AVF failure. As shown in Figure 1, we found a strong association (AUC: 0.814, *p* < 0.001), with an optimal cut-off value of 7.08 (76.5% Sensitivity and 79.7% Specificity).

**Figure 2:**
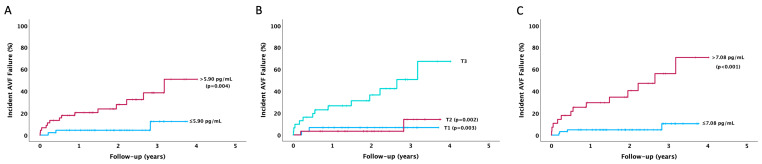
Kaplan–Meier survival curve: the association between IL-6 values at baseline and the incidence of AVF failure during the follow-up according to (**A**) median value, (**B**) tertiles value, and (**C**) optimal cut-off value.

Furthermore, at the survival curve Kaplan–Meier analysis, we observed a higher occurrence of AVF failure in patients with baseline IL-6 values above the median (*p* = 0.004) (Figure 2A), as well as in patients with IL-6 values in the third tertile compared to the first and second tertiles (*p* = 0.003 and *p* = 0.002) (Figure 2B). Moreover, as shown in Figure 2C, patients with preoperative IL-6 values higher than the optimal cut-off value face a significantly increased risk of AVF failure (*p* < 0.001).

In order to assess the predictive significance of the variables with significant statistical variances between the two groups of patients and the predictive role of baseline IL-6 values, we conducted a Cox regression analysis. Table 2 shows that the presence of a history of malignancy (HR: 4.25, *p* = 0.026) and active smoking (HR: 2.94, *p* = 0.036) are linked to long-term AVF failure. Furthermore, elevated baseline IL-6 levels are associated with AVF failure (HR: 2.23, *p* < 0.001), regardless of age and sex (HR: 2.18, *p* < 0.001), and independent of age, sex, and cardiovascular risk factors (DM, hypertension, IHD, and PAD) (HR: 1.96, *p* = 0.001). Furthermore, higher baseline values of IL-6 are associated with AVF failure in RC-AVF patients (HR:4.06, *p* = 0.001) and BC-AVF patients (HR:2.33, *p* = 0.012) (Table 2).

## 4. Discussion

The main finding of this study is that elevated baseline levels of IL-6 are linked to long-term AVF failure, regardless of age, sex, and cardiovascular risk factors such as DM, hypertension, IHD, and PAD. Additionally, a history of malignancy and the presence of active smoking is associated with vascular access dysfunction. Furthermore, patients who experience AVF failure during the follow-up period have higher levels of WBC, glucose, and platelets.

Numerous studies have investigated the link between systemic inflammation and AVF failure, focusing on biomarkers like IL-6 and CRP [14,16,17,18] or systemic inflammatory biomarkers derived from the total number of neutrophils, monocytes, lymphocytes, or platelets [11,19,20,21,22,23,24,25]. Kaygin et al. [16] found that out of 311 patients with successful AVF and 75 with unsuccessful ones, those with failed AVF had lower albumin levels, higher CRP, and fibrinogen levels (for all *p* < 0.001). Similarly, Chang et al. [14] discovered that the levels of IL-6 and TNF-α were significantly elevated in the thrombosed AVF group (*p* = 0.010 and *p* < 0.001).

Moreover, it is well-known that IL-6 initiates the inflammatory response by activating endothelial cells and fibroblasts, inducing the proliferation of smooth muscle cells, leading to intimal hyperplasia within the vessel, and enhancing vascular wall hypertrophy [17]. In a recent article by Kaller et al. [10], the authors found higher levels of IL-6 in patients with intimal hyperplasia in the venous wall at the time of the AVF creation (*p* = 0.0001). Additionally, the authors observed a strong positive correlation between IL-6 levels and the surface area positive for CD31 in the thickened intima (r = 0.611, *p* < 0.001) [10]. The findings mentioned are also present in other research papers, such as the study by Bi-Cheng et al. [18]. In their article, the authors found that patients with vascular access dysfunction have higher levels of IL-6 compared with patients with new AVF (*p* < 0.01) or patients with old well-functional vascular access (*p* < 0.05). Moreover, the authors found that patients with AVF failure had a thicker internal layer of vessels compared to those with new AVF (*p* < 0.01) [18].

Martínez et al. [26] analyzed the role of inflammatory cytokines in AVF maturation failure in a case-control study. They enrolled 64 patients and found higher levels of IL-6 in the group of patients with AVF maturation failure (*p* = 0.038). Additionally, Panichi et al. [27] showed that high levels of IL-6 are linked to long-term mortality in a cohort of 218 hemodialysis patients. IL-6 was found to be a stronger predictor of mortality than CRP. Similar to our study, but with a shorter follow-up period of only 12 months, Baek et al. [13] demonstrated that patients with high levels of IL-6 in tertile 3 are associated with AVF failure.

Our study has several limitations that need to be addressed. Firstly, it was a single-center retrospective study with a small number of patients enrolled. Therefore, in the future, our findings should be confirmed through multi-center prospective studies to determine an optimal cut-off value. Secondly, we only measured IL-6 levels pre-operatively and not their changes over time, which are important for analyzing long-term vascular access dysfunction. Additionally, we did not perform ultrasound evaluations during follow-up to assess the degree of intimal hyperplasia, which could have helped in understanding the relationship between baseline IL-6 levels and the severity of intimal hyperplasia. Lastly, due to the study’s retrospective nature, we lacked information regarding the relevant medications, arterial flow, and post-operative AVF debit from the hospital’s electronic database.

## 5. Conclusions

In the current study, we demonstrated that elevated IL-6 levels at baseline are associated with long-term AVF failure, independent of age, sex, and cardiovascular risk factors such as DM, hypertension, IHD, and PAD. Moreover, the predictive role of IL-6 on AVF failure is also confirmed in the subgroup of patients with RC-AVF and BC-AVF. Additionally, a history of malignancy and active smoking is associated with vascular access dysfunction. Furthermore, patients who experience AVF failure during the follow-up period have higher levels of WBC, glucose, and platelets. Although the measurement of IL-6 involves higher costs than the routine biomarkers, its determination could provide a new tool to predict arteriovenous fistula failure and develop a new therapeutic strategy to inhibit IL-6 and intimal hyperplasia development.

## Figures and Tables

**Table 1:** All the characteristics of the patients enrolled in the study, presented based on AVF failure during the follow-up.

Variables	All Patients n = 91	Functional AVF n = 74	AVF Failure n = 17	*p* Value
Age mean ± SD	62.93 ± 13.28	62.97 ± 13.28	62.76 ± 13.68	0.955
Male gender no. (%)	49 (53.85%)	42 (56.76%)	7 (41.18%)	0.245
**Comorbidities and Risk factors, no. (%)**				
Hypertension	83 (91.21%)	68 (91.89%)	15 (88.24%)	0.631
Atrial Fibrillation	11 (12.09%)	8 (10.81%)	3 (17.65%)	0.436
Diabetes Mellitus	36 (39.56%)	25 (33.78%)	11 (64.71%)	**0.019**
Ischemic Heart Disease	58 (63.74%)	45 (60.81%)	13 (76.47%)	0.226
Peripheral Arterial Disease	17 (18.68%)	12 (16.22%)	5 (29.41%)	0.208
History of Malignancy	7 (7.69%)	4 (5.41%)	3 (17.65%)	0.088
History of Myocardial Infarction	7 (7.69%)	5 (6.76%)	2 (11.76%)	0.485
History of Stroke	6 (6.59%)	5 (6.76%)	1 (5.88%)	0.895
Active Smoking	14 (15.38%)	8 (10.81%)	6 (35.29%)	**0.012**
Obesity	23 (25.27%)	19 (25.68%)	4 (25.53%)	0.854
**Laboratory data, median (Q1–Q3)**				
WBC	7.81 (6.34–9.51)	7.20 (6.14–9.02)	9.40 (8.19–10.60)	**0.008**
Potassium mmol/L	5.18 (4.66–5.65)	5.13 (4.59–5.55)	5.23 (4.86–6.03)	0.075
Sodium mmol/L	139 (137–141)	140 (137–141)	139 (138–140)	0.674
Glucose (mg/dL)	102.50 (89–136.22)	97.60 (88–126.90)	128.90 (106–192.5)	**0.007**
BUN (mg/dL)	134.76 (90.8–168.73)	134.82 (93.4–167.1)	123.90 (90.5–174)	0.945
Creatinine (mg/dL)	6.69 (5.50–8.81)	6.72 (5.41–9.09)	6.65 (5.85–7.55)	0.909
Hemoglobin g/dL	10.60 (9.21–11.4)	10.47 (8.7–11.25)	10.60 (10.10–11.80)	0.158
Hematocrit %	31.72 (28.5–35.21)	31.66 (27.57–35.07)	32.30 (30.30–36.10)	0.269
Neutrophils ×10³/µL	5.20 (4.17–6.54)	4.95 (4.15–6.30)	6.68 (4.86–8.5)	**0.023**
Lymphocytes ×10³/µL	1.41 (1.11–1.93)	1.41 (1.09–2)	1.35 (1.22–1.81)	0.891
Monocyte ×10³/µL	0.58 (0.445–0.77)	0.57 (0.42–0.76)	0.69 (0.57–0.77)	0.132
PLT ×10³/µL	211 (171.50–281.50)	201.5 (168.25–272)	245 (211–304)	**0.048**
Interleukin-6 pg/mL	5.91 (4.71–7.61)	5.70 (4.47–6.97)	8.29 (6.11–11.4)	**<0.001**
**Vascular Mapping Determinations, median (Q1–Q3)**				
Arterial Diameter (mm)	2.80 (2.27–3.50)	2.95 (2.30–3.50)	2.35 (2–4.50)	0.353
Vein Diameter (mm)	3 (2.40–3.50)	3.10 (2.42–3.50)	2.50 (2.40–3)	0.143
Vein Depth (mm)	2.50 (1.99–3.50)	2.50 (2–3.50)	2.00 (1.80–3.40)	0.430
**AVF Type and Placement, no. (%)**				
RC-AVF	41 (45.05%)	31 (41.89%)	10 (58.82%)	0.206
BC-AVF	39 (42.86%)	34 (45.95%)	5 (29.41%)	0.214
BB-AVF	11 (12.09%)	9 (12.16%)	2 (11.76%)	0.964
Dominant Upper Limb	15 (16.48%)	12 (16.22%)	3 (17.65%)	0.886
Dialysis on CVC at the time of AVF creation, no. (%)	53 (58.24%)	46 (62.16%)	7 (41.18%)	0.114
Follow-up period (years) mean ± SD	1.53 ± 0.94	1.67 ± 0.89	0.91 ± 0.93	**0.002**

The *p* Values highlighted in bold indicate statistically significant differences (*p* < 0.05).

**Table 2:** Cox regression analysis: the association of comorbidities and IL-6 value at baseline and AVF failure during follow-up.

Variables		AVF Failure		
		HR *	95% CI	*p* Value
Diabetes Mellitus		2.69	0.99–7.31	0.051
History of Malignancy		4.25	1.19–15.21	**0.026**
Active Smoking		2.94	1.07–8.06	**0.036**
IL-6	Model 1	2.23	1.57–3.18	**<0.001**
	Model 2	2.18	1.53–3.12	**<0.001**
	Model 3	1.96	1.31–2.97	**0.001**
IL-6—RC-AVF	Model 1	4.06	1.73–9.52	**0.001**
IL-6—BC-AVF	Model 1	2.33	1.21–4.51	**0.012**

* HR expressed per 1 SD increase in baseline IL-6; Model 1: unadjusted; Model 2: age and sex; Model 3: age, sex, and CV risk factors (diabetes mellitus, hypertension, ischemic heart disease, peripheral arterial disease). The *p* Values highlighted in bold indicate statistically significant differences (*p* < 0.05).

## Data Availability

The data that support the findings of this study are available from the corresponding author upon reasonable request.
